# Paradox of the institution: findings from a hospital labour ward ethnography

**DOI:** 10.1186/s12884-016-1193-4

**Published:** 2017-01-03

**Authors:** Elizabeth C Newnham, Lois V McKellar, Jan I Pincombe

**Affiliations:** School of Nursing and Midwifery, University of South Australia, GPO Box 2471, Adelaide, South Australia 5001 Australia

**Keywords:** Childbirth, Ethnography, Epidural analgesia, Foucault, Medicalisation, Medical anthropology, Midwifery, Surveillance

## Abstract

**Background:**

Interest in the influence of culture on birth practices is on the rise, and with it comes a sense of urgency to implement practices that aid the normalisation and humanisation of birth. This groundswell is occurring despite a broader cultural milieu of escalating technology-use and medicalisation of birth across the globe. Against this background, rates of epidural analgesia use by women in labour are increasing, despite the risk of side effects. Socio-cultural norms and beliefs are likely to influence pain relief choices but there is currently scant research on this topic.

**Methods:**

This study was undertaken to gain insight into the personal, social, cultural and institutional influences on women in deciding whether or not to use epidural analgesia in labour. The study had an ethnographic approach within a theoretical framework of Critical Medical Anthropology (CMA), Foucauldian and feminist theory. Given the nature of ethnographic research, it was assumed that using the subject of epidural analgesia to gain insight into Western birth practices could illuminate broader cultural ideals and that the epidural itself may not remain the focus of the research.

**Results:**

Findings from the study showed how institutional surveillance, symbolised by the *Journey Board* led to an institutional momentum that in its attempt to keep women safe actually introduced new areas of risk, a situation which we named the *Paradox of the institution*.

**Conclusions:**

These findings, showing a risk/safety paradox at the centre of institutionalised birth, add a qualitative dimension to the growing number of quantitative studies asserting that acute medical settings can be detrimental to normal birth practices and outcomes.

## Background

Understanding of the influence of cultural beliefs on women’s experiences of labour and birth has been increasing since Brigitte Jordan’s seminal text *Birth in Four Cultures* [1] first explored the ways in which cultural expectations affect both the care given, and women’s approach to birth. Such texts illustrate how wider cultural norms and accepted understandings of childbirth, technology and medical expertise help to shape not only women’s knowledge of the birth process, but also their attitudes towards their bodies, their babies, and their birth experiences [2]. While culture is known to have an impact on birth understandings and practices [1, 3, 4], there is less research on the impact of culture specifically on the choice to use analgesia.

The use of epidural analgesia in labour continues to increase despite the fact that epidural use carries risks such as hypotension, longer second stage of labour, increased instrumental birth and decreased breastfeeding rates [5–14]. Walsh suggests that rising epidural rates are influenced by increasing technocratic values within a fragmented maternity system that leaves women feeling unsupported, alienated and frightened [15]. Interested in examining this problem from a cultural perspective, the focus of our research was therefore to examine meanings, practices, and choices in childbirth within a public hospital, by investigating women’s use of epidural analgesia in labour. The research was undertaken with attention to wider social influences, such as the increasing value of, and reliance on, technology in the late capitalist economy, and the dominance of scientific discourse and its impact on gender constitution in general, and childbirth rituals in particular.

**Fig. 1:**
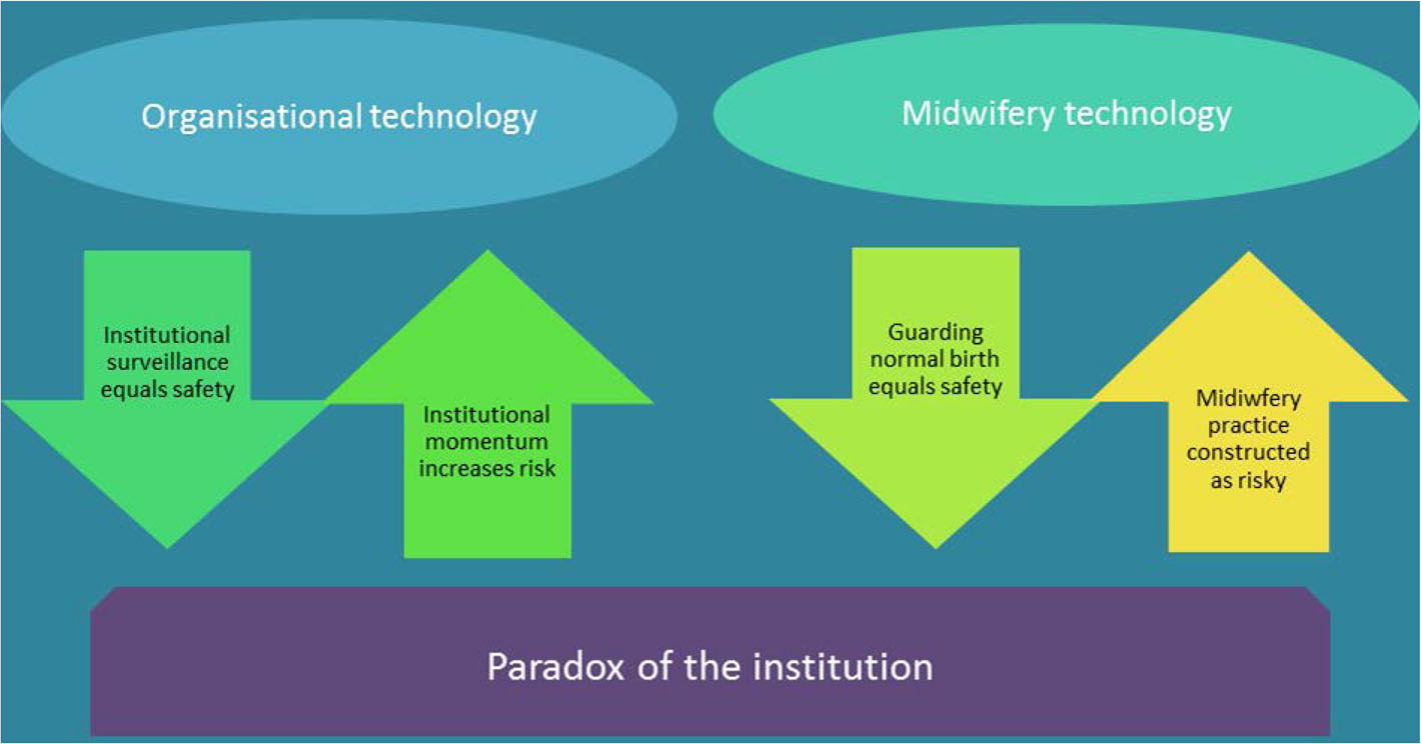
Paradox of the institution

In this article, we use an intermediate social level of analysis to describe the dynamics of the hospital [16]. After a brief overview whereby we position the institution from the perspective of a critical methodology (CMA), we outline Foucault’s identification of the medical gaze, and depict how institutional surveillance, the *Organisational technology*, was symbolised by the *Journey Board*. The *Institutional momentum* is then described, underscored by the concept of *Time in labour* and the ways in which practitioners worked within or resisted these temporal constraints. These themes are drawn together in the discussion to form one half of the concept of the *Paradox of the institution* (Fig. 1). Space does not allow for discussion of the other half of the Paradox, the *Midwifery technology*, which will therefore be addressed in a separate paper.

## Methods

This study is situated within a growing body of midwifery research that continues to promote ‘normal birth’ [17–19], as well as contributing to sociological and anthropological theories about birth and motherhood [3, 20–23]. EN, supervised by LM and JP, used an ethnographic methodology, underpinned by Critical Medical Anthropology (CMA) and supported by Foucauldian and feminist theory, to critically examine routine epidural use by exploring the personal, social, cultural and institutional influences on women in deciding whether or not to use epidural analgesia in labour. Ethnographic research typically has a broad focus, and does not tend to specify a research question. Therefore, although increasing epidural analgesia uptake was the primary research problem, given the nature of ethnographic research as the study of culture, it was anticipated that epidural use would form part of a greater picture of birth culture, and may not, in fact, remain centre stage. From this perspective, EN examined concepts of birth pain and its relief, with a particular focus on the institution as an arbiter between the macro—socio-political norms— and the micro—individual decision-making and interactions.

In this article, we explore the data from the labour ward observation and using an intermediate social level of analysis which centres on institutional policy and decision-making processes (p. 96) [24], and how clinicians interact with these, as well as with each other, within the milieu of the institution. This analysis therefore focuses on the dominant cultural beliefs and practices within the institution—which centred primarily on risk management and client throughput—and the ways in which midwives and other clinicians articulated, negotiated, or resisted these norms, as well as the disciplinary mechanisms that were used to promote and uphold them.

### Setting

In 2012, EN conducted participant observation fieldwork in a large, urban, tertiary hospital in an Australian capital city. The hospital has a large catchment and is the primary major hospital for the local area. EN attended the hospital labour ward (obstetric unit) for approximately two days a week, across all shifts, for a period of six months. As with many labour wards, there was a central midwives’ station and a number of labour rooms. No further detail is provided here in order to preserve setting anonymity.

### Data Collection

Data collection consisted primarily of participant observation, which included documenting informal discussions with hospital midwives and doctors, as well as observation of practices and conversations, into comprehensive field notes. Sixteen pregnant women were also recruited from the hospital antenatal clinic to participate in a series of sequential interviews; two antenatally and one postnatally. EN attended the labour and birth of six of these women as an observer. A third element, hospital and policy document analysis was incorporated into the ethnographic study to contribute to data triangulation [25].

### Reflexivity

EN was aware that investigating increasing epidural use as a problem was a potential source of bias. In fact, this bias was made explicit, in EN’s position as a feminist, a mother of four, and a midwife with a belief in the necessity of ‘keeping birth normal’ and the experience of birth as a powerful physiological event that has ongoing social, sexual and psychological effects on women’s lives. Understanding one’s position is fundamental to the location of the frameworks of power in critical research [26–28]. In this way, EN maintained a reflexive position, journaling thoughts and identifying potential analytic bias, repeatedly returning to the data, and following up discongruencies, which can lead to deeper research insights [28, 29]. Following Geertz [30], EN also strove to provide thoughtful and detailed interpretation; ‘thick description’, strengthened by the theoretical underpinning of CMA.

### Data analysis and research rigour

Data, therefore, were analysed using Geertz’ concept of ‘thick description’, as well as an analytic framework adapted from Baer, Singer and Johnsen’s *Levels of Health Care Systems* model that frames data analysis according to four system-levels, from the macro to the individual perspective [16, 24]. Using this analytic method, underpinned by the triad of chosen theory, we identified ways in which the experience of birth have been shaped by hegemonic discourses, with a view to recognizing how these understandings have been embodied or resisted by women and midwives.

Geertz argues that looking at specific cultural practices does not necessarily give rise to an understanding of the whole, but that ethnographic interpretation is ‘tracing the curve of a social discourse; fixing it into an inspectable form’ (p. 18) [30]; that is, ethnography provides meaning not truth. If we accept this premise then the application of the research also changes [26, 31]. Rather than being generalisable to the population, the idea of ‘fittingness’—in which the findings of qualitative research studies are seen to ‘fit’ with the experience and meaning-making of readers and therefore applicable to other situations or locations—has been proposed [32]. Australia, as a Western, developed nation has many things in common with other, similar nations, including medicalised birthing practices within hospitals. This is where the informal peer-review of conference presentations was fruitful; the findings were clearly resonating with other midwives in other countries—they were not simply a figment of one researcher’s solitary trawl through the data, which also increases the rigour of the study. EN also compared the findings to theory and other studies in the field, reinforcing the fittingness of the study as similarities were drawn.

### Theoretical perspectives: The institution and the ‘medical gaze’

Institutional beliefs and practices have an impact on the choices that people within them make. Political economy of health theorists have critiqued institutionalised health care in various ways, including describing institutions not as neutral, but as places wherein power relationships are reproduced and maintained [33]. Seen in this light, institutions therefore have the potential to recreate medical dominance over the birth process. Women, birth, midwives and midwifery practice have long been at the centre of interactions of power, some of which continue, and which drove, at least in part, the relocation of birth from home to the hospital [34–36]. Health care institutions have been identified as alienating places, for both patients and health care workers, due to increasing fragmentation and reliance on specialisation and technological expertise [37]. However, in focusing on technologies, the individual person can be lost, and practices can become based on institutional needs rather than the needs of the person. Despite increasing knowledge about what can potentially halt women’s labours, such as noise, intrusion, light, and anxiety [22, 38], medicine will still seek a physical—a mechanical—reason for labour dystocia (p. 62) [22], exemplifying biomedicine’s focus on the mechanical body, the ubiquity of an industrial measurement of time and a reliance on technological intervention [39–41].

According to Foucault, hospitals emerged as a plentiful source of material for medical students and practitioners alike to examine bodies and gather information (p. 102) [35]. In addition, hospitals were increasingly regarded as abundant sites of information for the growing government interest in the body—both individual and social [35, 42]. The decreasing use of explicit force or coercion and increasing surveillance and regulation of bodies by government is termed by Foucault ‘disciplinary power’ (p. 140) [42]. This dual function of hospitals therefore served to reinforce them as ‘apparatuses of surveillance’ (p. 101) [43], offering subjects for the ‘medical gaze’ (p. 67) [44]. During field work, EN noted down how the ward was organized, where events occurred, and also much of the dialogue—both that said to her and in front of her. She began to notice the deeply entrenched surveillance that occurred in the ward, and the disciplinary regulation that occurred. Disciplinary power was exerted in the architecture of the rooms and how spaces were used, it was evident in the time impositions on women in labour, and the pressure that midwives were under to ‘make time’ for women, and it was particularly evident in the primary mechanism of surveillance, the hospital patient *Journey Board* (Table 1).

**Table 1:** The Journey Board

Bed No	IOL/SOL LSCS	Gravida/parity	Gest	Membranes	Cx	Next exam	CTG	Synt	Analgesia
				Time/intact/colour					
				SRM					
				ARM					

### Findings

#### Organizational technology: The Journey Board as surveillance

The *Journey Board* was a large whiteboard in a central location behind the midwives’ station and included information such as: women’s names, status (in labour, antenatal, postnatal), and if in labour, cervical dilatation at what time, when next examination was due, and any review needed. It operated as a mechanism of surveillance in that it not only offered a source of continuous information about the women themselves, but also an overview of the unit more broadly, in terms of staffing needs and bed requirements.

*The board is displayed at the back of the midwives’ desk… Up the top is the shift coordinator’s name, and a key of symbols,* e.g. *‘seen’, ‘needs to be seen’, ‘CTG [cardiotocograph]’,* etc. *At the left hand side is the midwife allocated to care for the woman, the bed number, then the woman’s full name, type of labour/birth (IOL [induction of labour], SOL [spontaneous onset of labour], LSCS [lower segment caesarean section]), gestation, membranes (time, intact, ARM [artificial rupture of membranes], SRM [spontaneous rupture of membranes], colour), Cx [cervix] (dilatation and time), next exam due, CTG (yes/no), GBS [group B streptococcus] status, Group & Save (yes/no), Syntocinon, analgesia, remarks (*e.g. *postdates, allergies, GD [gestational diabetes], Rh neg). (Field notes 2/5/12).*

The use of different colour markers signified if a woman was antenatal, in labour, or postnatal. When a woman had given birth, the board was marked with a (D), indicating that she had ‘Delivered’. Table 1 is representative of the *Journey Board*, although not an exact reproduction so as to maintain confidentiality of the institution.

The *Journey Board* is reminiscent of Foucault’s Panopticon—his symbolic description of the way in which people are encouraged to conform simply because of the possibility of being observed, even if they are not under direct observation (p. 200) [45]. The disciplinary power invoked by this model, based on Bentham’s design for a penitentiary, is founded on indirect observation—for example, the collection of information—and power exerted by manipulation of this knowledge, rather than direct coercion (p. 214–15) [45]. The strength of disciplinary power, Foucault argues, is precisely that it requires no coercion, as the techniques of power utilised encourage self-surveillance and self-regulation—where individuals mark and discipline their behaviour according to a set of implicit social norms [45]. Thus, not only did the *Journey Board* have a normalising effect on the progress of women’s labour, its function was also in disciplining midwives and doctors in their work. The next two excerpts show how the midwives talked about the effects of this surveillance.

*The doctors stand there and look at the board. If they’re 3 cm [dilated] they want them to be seven in four hours. If they [women] don’t [dilate fast enough], then they start talking about ARM, Synt (MW2).*

*I hate examining them. You know, ‘cause once they’re fully [dilated], then the doctors put a time limit on. The registrars, I know most of them, and they trust my judgement, but then they report to the consultants who don’t know the women, and want everything to follow a protocol (MW4).*

The pressure of ‘observation’ was a recurring theme in the midwives’ talk. Conversely, in Walsh’s ethnography of a free-standing birth centre [46], he found that the lack of external and medical monitoring was a positive influence on the midwives, stating:

The reality that escaping surveillance may facilitate non bureaucratic ways of achieving goals reinforces Foucault’s concept of panopticism and its constraining effects. By being outside the ‘gaze’, the staff experienced a freedom that, for them, was extremely creative (p. 1336) [46].

Our findings support those by Walsh, as they illuminate the contrast between these models of practice, and identify the specific ways in which medical surveillance curbed and restricted midwifery practices and the space and time in which women laboured.

The *Journey Board* was thus both a mechanism of surveillance of individual women and their progress through labour, as well as the practices of the midwives looking after them, and also worked as an organizational tool for managing the ward and hospital, shown by the following field note excerpt.

*The ‘board’ appears to be the main focus of attention in the ward. Team leaders, doctors, midwives who come out to the desk, periodically throughout the shift, come and stare at the board. There is discussion about who is doing what, who is coming in, how long they will be here for* etc. *It seems to be a process of organising…Discussion at the board between T/L [shift team leader] and clinical midwife: ‘Let’s try and get one delivered, then we’ll be ok [staffing and bed numbers]. We’ll target room seven’ (Field notes 2/5/12).*

The language in the above excerpt shows an institution-focused, rather than woman-focused mentality; a focus which is known to disrupt the midwife-woman relationship and is unsuited to midwifery philosophies and humanized birth practices [4, 47–50]. Despite this, there were also many examples of midwives giving woman-centred care within these institutional parameters. In fact, one of the main drivers for being institution-focused was to process women through the system quickly, with the aim of avoiding the situation where everyone is unsafe because of the unit becoming over-full.

*I spoke to a midwife who was on the late [shift] last night. After I left it got messy. They had two MET [Medical emergency team] calls. One for the woman who came in by ambulance yesterday. She had a fainting episode, and ‘looked like she was abrupting.’ The other [MET call was for] a woman with a massive PPH. These high-risk episodes are surely the kinds of events that cause the ‘risk aversion’ behaviours previously mentioned (Field notes 21/6/12).*

While the risk focus of the institution meant that emergencies and complications were managed well, with high levels of clinical expertise, the view through the lens of this research framework brought another perspective. It was as if the institution itself was trying to cast women out in order to keep them safe; as if the longer the women are there, the more likely an error or an intervention or a staffing issue will occur; as if the risk discourse of the institution is a self-fulfilling prophesy.

*I have been thinking about the staffing issues, and how they affect the way that things happen here. They don’t want women here who don’t need to be here (eg early labourers) and like to keep things moving along simply so that the labour ward doesn’t fill up. It is a safety issue (Field notes 21/6/12).*

Hunt and Symonds, in their landmark ethnographic study of midwifery, also observed that women in early labour (‘nigglers’) would be moved from the labour ward if possible [51]. This was in part because they did not denote the ‘real work’ of midwifery on the labour ward, but it was also an issue of bed blockage (pp. 98–104) [51]. The following excerpt describes the reality of bed-blockage, highlighting the kinds of unsafe scenarios for which the institutional momentum existed to avoid.

*The board is full. Two women of 30 weeks gestation are in labour, and another woman who is 35 weeks. There is a woman being induced for epilepsy. Two postnatal women on MgSO4 for pre-eclampsia, there are two women having elective CS, and one woman due to have a CS for two previous CS has come in contracting. Another woman who is scheduled for a CS later in pregnancy with twins has come in with a query of ruptured membranes. The T/L goes through it all with the consultant, then, as there is a mix up with who is covering labour ward that day, goes through it all again with a registrar. If someone else comes in labour, there will be no bed for her (Field notes 11/7/12).*

Discourses of both risk and safety therefore provide an impetus for moving women through the system. The hospital environment has been fashioned as the predominant site of safety for birth, and yet it behaves as a site of risk. It is structured to process women quickly in order to mitigate further risk of being held up in—or holding up—the institution, and to keep women ‘safe’ by discharging them. The aim of keeping women safe within the institution was a very real concern for midwives and the obstetric unit fulfilled its function in mitigating risk in the presence of emergencies. However, rather than viewing the ‘processing’ of all women through this system as an appropriate solution, we suggest instead, following Dykes [52], that the situation we describe confirms the unsuitability of large, acute, medical institutions as the appropriate site for all birth.

As well as being a surveillance and organizational tool for managing the ward, The *Journey Board* was used as an obstetric management tool.

*As the night progresses, the monitoring system is observed, the board attended to, updates given. T/L: ‘The woman in Rm four is fully, at spines, should have a baby in there soon.’ Obstetric registrar ‘Oh good’ [pause] ‘The trace looks beautiful doesn’t it?’ (Field notes 31/5/12).*

The *Journey Board* thus represented the functionality of the unit as a whole, not only providing a system of organising staffing and bed numbers, but a visual representation of a ‘risky’ or ‘safe’ ward depending on the colour scheme. The following comment by a registrar typifies the general relief felt when women have passed successfully though the labour process—as this is where the risk of emergency is greatest—and are now written on the board in green.

*[There is a] registrar standing at the board with [a group of] new students or RMOs explaining how it works: ‘Green is postnatal. They don’t cause much trouble, so I like to see a Green board’ (Field notes 2/5/12).*

With a ‘green board’, not only can the doctors relax, as an emergency is now unlikely, it also signals a ‘green light’ for the unit, as these women can be moved to the postnatal floor or discharged home. This sense of relief is also an example of how pervasive surveillance within a risk culture works equally on doctors and midwives, and is not necessarily imposed by obstetricians themselves, but by these systems of power/knowledge and their influence on the institution, as well as practice, that have been produced in relation to medicine, midwifery and birth [53, 54].

The centrality of ‘the board’ in monitoring and managing labour progress and organising practice has been mentioned in other studies undertaken in the UK [51, 55], though without a specific focus on ‘surveillance’ from a Foucauldian perspective. The appearance of ‘the board’ in other studies lends credence and validation to these findings’ ‘fittingness’. The disciplinary power symbolised by the *Journey Board* in this study was pervasive throughout the unit. A senior labour ward midwife was alerted to this after attending a conference where an independent midwife had given a talk about a ‘midwifery approach’ to labour. Sometime after this, at work, while assessing a woman by vaginal examination, the midwife conducted an artificial rupture of membranes (ARM) because she ‘*knew ‘they’ would want it anyway*’ (in order to see the liquor colour, and hasten birth). However she was now reflecting on and questioning her practice, in part because it had ended up as a ventouse birth. This is interesting not only because it illustrates the disembodied, panoptic surveillance that was so pervasive that the midwife did not need anyone to tell her directly to do an ARM, she simply knew that ‘they’ would want one, but—and perhaps more importantly—she only reflected on this practice after being exposed to a less intrusive philosophy of midwifery. The impact of this surveillance on midwifery practice is important and requires further investigation. One study has identified how senior labour ward midwives carried checked up on progress 2-hourly because ‘the doctors expect it’, despite it not being hospital policy, nor actually expected by the doctors [55]. It has been suggested that midwives, dealing with the dissonance between the ideal of woman-centred care and the reality of institutional birth, have externalized responsibility to the extent that they conform to medicalised practices even when the perceived barriers are not there [56]. Using the notion of authenticity, midwives can choose between a range of possible practice options, increasing potential responses and possibilities for woman-centred practices [56]. With awareness of this and use of evidence, it is an area where midwives could now potentially exert some influence in normalising birth.

The surveillance and organizational role of the *Journey Board* is perhaps understandable in large hospitals where they are used in part to prevent errors and near misses from occurring, and therefore work as a safety mechanism [57, 58]. However, although this mechanism may work extremely well for medical or surgical patients, it is arguably not suited for monitoring labouring women. In fact, O’Brien, Bassham and Lewis (p. 161) discuss how they ‘were also influenced by models used in an industrial setting’ and cite *The Toyota Way*, a field book produced by the Toyota car manufacturing company [58]. Arguably, a safety mechanism designed on industrial processing is not going to benefit women undergoing labour and birth; a fluid, psychophysiological human process.

### Playing for time

While some midwives appeared quite comfortable working within the institutional culture, others felt it interfered with their own ideas of what it means to be a midwife, and expressed frustration at the intense pressure from the institution to work within a time frame that was external and artificial rather than working with individual women’s rhythms of labour. Many of the midwives in this study discussed their impressions that they were continually pushing against the rigid parameters of time engaged by the institution.

*The most frustrating thing about working here is you just want to slow everything down. I mean, just give her a chance, you know? (MW50).*

*I do a lot of nights because then I can just do my job. It’s hard because you have to fit into the institutional constraints (MW4).*

*It’s frustrating you know, day in, day out. It depends on the doctor. Some are just worse than others. They jump in too quickly. They don’t give them a chance (MW43).*

Accounts by independently practicing midwives describe wide variation in labour patterns where midwives use a watchful approach toward the unpredictable nature of birth rather than one of control—for example, a woman at home might stop labouring and sleep for a few hours, then wake up refreshed and push out her baby [59, 60]. In a hospital, this same labour, under the ‘medical gaze’ is likely to be augmented, leading to an increasing need for analgesia and intervention, as the institution imposes an externalized and artificial timeline on a process that is individually and uniquely experienced [54, 61].

*The other thing I’ve noticed is they do an ARM here, and they’ll want to put Synt up in the next hour. And I’m like ‘Why?’ But no-one gives me an answer. I have a feeling it’s about beds, but that’s not right. That woman deserves to be given that space for labour. If we do an ARM, she’s most likely going to go into labour, she just needs time (MW34).*

Despite the fact that ‘Friedman’s curve’—the template for documenting progress of labour by measurement of the cervix—is based on inadequate and outdated research and there is no real evidence on what is a ‘normal’ length of labour [19, 54], there was an institutional demand to ‘push women through’ the system. Some midwives worked against the institutional momentum. In addition, some doctors would also attempt to give women more time, as shown in the following field note excerpt.

*A midwife comes out to discuss the progress of the woman she is caring for in labour with the registrar. She wants to know whether or not Syntocinon will be required and if so, when. The woman has had an epidural in since 9 am. The midwife has done a vaginal examination (VE) and the woman is 7–8 cms dilated. There is discussion about the contractions. The midwife explains they feel a little less strong than before the epidural but still 3: 10.*

*Moderate? Asks the registrar.*

*Midwife35: Umm yep.*

*Registrar: Well, she is a primip, so…let’s give her some more time if she is contracting well.*

*Midwife35: So when shall I reassess her?*

*Registrar: Well, in four hours.*

*Midwife35: Ok, great [lets T/L know].*

*T/L (to registrar): Good decision.*

However, 2 h later, during afternoon handover, the consultant came around, and wanted an earlier assessment:

*The consultant came and overrode the registrar’s decision and wanted a VE in 2 h (from the 7–8 cms).*

*T/L: Oh, she’s written ‘consider’ here [ie consider another VE in 2 h] as a compromise. But Dr [consultant] comes in and she wants, you know, it all to happen, to be fully in 2 h (Field notes 11/7/12).*

This excerpt shows how at times both doctors and midwives worked together to provide the time and space for women to birth. The midwife is checking to see if the doctor would want to order Syntocinon, given that the woman has an epidural and her contractions are slowing. Although the midwife would have obliged by putting up a Syntocinon drip had the doctor ordered it, she was pleased when the doctor did not. The T/L also reinforced the doctor’s decision by giving positive feedback. In this way, inroads into interdisciplinary collaboration were made, with potential benefits to the women, even though, in this case, the attempt to ‘buy’ the woman more time was overridden by the consultant obstetrician.

Notably, beneath the surveillance of the *Journey Board*, there was often interdisciplinary collaboration to buy women more time, which is a positive finding and highlights an area that could be further developed with appropriate knowledge and training of normal birth practices. It is also a place to begin changing the system from within.

### Institutional momentum

Figure 1 illustrates the organisation of data that is explored in this paper, showing how the competing technologies of practice culminated in a paradox of the institution.

In order for women to fit within the demands of the institution to run like clockwork, EN noticed practices that served to ‘push’ women through the system. The following excerpts show some examples of this, as well as her written reflections at the time, which contributed to the subsequent analysis.

*8 am doctors round [standing at board]. The consultant is pleased that the registrar has begun the inductions. Registrar: ‘Well, that’s what we used to do at hospital X. ARM and Syntoed [commenced a Syntocinon infusion] them all overnight [early hours of morning] and then they’d all be ‘going’ when the morning staff came on’ (Field notes 10/5/12).*

*This accentuates the difference in philosophy between midwifery and medicine around birth. There was an understanding between these two doctors that a reduction in workload for the oncoming doctors was a good thing. It also keeps within the lines of the functioning hospital idea, where women left to their own devices are seen as displeasing to medical staff, as if they are making the place untidy (Analytic memo 10/5/12).*

This idea that the needs of the institution come before the needs of the woman has been raised by others [49, 62, 63]. Murphy-Lawless writes of the maternity hospital:

A key element in this organisation is keeping up the throughput of women and, whatever the rhetoric may be about individual choice, the bottom line is to ensure that the individual woman does not upset the system with her own demands or reactions to handling labour…Under such circumstances it is immensely useful to the obstetric system to draw on variants of its own historically grounded argument about the natural unreliability of the female body in labour (p. 42) [34].

This was certainly played out in the field site, where the pressure to continue the momentum of the institution meant that women were often pushed to keep up with the pace of institutional time rather being left to follow the rhythms of their labouring bodies. These bodies, the stalled labours, the augmentations and inductions, were then ‘fixed’ with interventions such as ARM and Syntocinon. The obligation to keep the institutional cogs in motion resulted in practices occurring purely because they fit within this institutional interventionist rhythm. In the field excerpt below, EN had been listening to a conversation between the T/L and a midwife looking after a woman who was being induced for diet-controlled gestational diabetes (GD). As they were looking up the state practice guidelines regarding blood sugar monitoring in labour, EN mentioned that women with diet-controlled GD sometimes had their babies in birth centre.

*‘Yes’, says the T/L, ‘I used to do that in birth centre, and we didn’t do hourly blood sugars’. In fact, this woman shouldn’t even be induced – that’s what the consultant said when she came on this morning: ‘Why is she being induced? It’s not necessary. She’s 39 weeks and has diet-controlled GD?’*

*T/L: ‘It would be different if she was on insulin, then she should be induced at 38 weeks. But oh well, it happens here all the time’ (Field notes 10/5/12).*

Our understanding of this last, throwaway comment by the midwife ‘*it happens here all the time*’ is that women get caught in the smoothly running institutional cogs, which do not stop rolling regardless. Thus, even when an irregularity is discovered—a woman who should not have been induced—the institutional apparatus rolls on regardless as if no-one has the power to stop it. This *Institutional momentum* is in fact another entity in the contested space of childbirth.

This next excerpt sums up the feeling when the unit is full, and in fact is an apt expression describing the underpinning cultural philosophy in general.

*In handover I hear: ‘She’s a time bomb waiting to happen. There’s no point sitting on her’ (Previous CS, scar dehiscence, other issues) (Field notes 25/7/12).*

And later on, that same phrase: *‘She’s just a little time bomb’ (Field notes 8/8/12).* There was an escalating sense of pressure that the midwives articulated: ‘*We’re all under pressure. We are all ready to explode. We are all stressed, all tired, we’re all feeling it’ (Field notes 15/8/12).* There was a definite sense in which women were ‘pushed through’ the labour ward, and that midwives also felt ‘pushed’ in trying to keep up in their efforts to avoid a full board and an unsafe unit. This contributed to a general feeling of building pressure; a metaphor which was applied to the women who they thought might ‘go off’ like a time bomb. Although ‘pushing women through’ the system arguably avoids the problems of understaffing, or bed-block, it ignores the knowledge that women’s labours have a unique rhythm. This rhythmic dissonance means the labouring woman is by definition ‘out of synch’ with the institution, and places her at risk of having the requirements of her body’s physiology ignored in lieu of reliance on technological intervention.

## Discussion

The lack of capacity within the labour ward (due largely to the effective surveillance engendered by the *Journey Board*) to ‘allow women time’ despite the attempts of various practitioners at ‘playing for time’, and the way in which the institution’s momentum propelled women into interventions even if they did not actually require them, is concerning, and poses questions about the safety of this system. This risk/safety contradiction (putting women at risk in order to maintain their safety) exposed a hidden paradox (Fig. 1) which was further compounded by observations about the midwifery role.

The centrality of the *Journey Board* meant that women were under constant surveillance and often placed within time constraints by maternity personnel who also needed to maintain the safety and efficiency of the institution—competing needs, against which the women often lost. However, the way in which people have perceived time has changed according to social and cultural understandings. With industrialization, there was a shift towards linear time as the clock became a symbol of the discipline of the factory’s ‘social relations of production’ (p. 100) [64] and factory rhythm replaced agrarian labour and cottage craft rhythm, which was more cyclical and seasonal [61, 64]. The change of location to the hospital as the primary site of birth echoed these wider social circumstances, such as increasing industrialization and factory work, and the corresponding reliance on the regular, systematic and linear management of time by the clock [65]. Martin equates the phases of labour (in birth) to a production line in a factory whereby deviation from normal ‘rates’ of labour equals disorder (p. 59) [22]. She observes that women, ‘grounded whether they like it or not in cyclical bodily experiences, live both the time of industrial society and another kind of time that is often incompatible with the first’ (p. 198) [22]. The freedom of organising time in a rhythmical or cyclical way was lost with the changes in the time measurement, and this was apparent in the comments of the midwives in the study.

Stevens observed that although reliance on clock time, including ordering and monitoring labour progress by this mechanism, served to ‘create order out of chaotic situations’ and give the impression of ‘efficiency’, it ultimately prohibited any kind of individualized or unique expression of time in labour (p. 111) [55]. Despite a philosophy of ‘being with’ women, institutional demands and a focus on linear time have been shown to impact midwifery practice [52], sparking a tendency to value being ‘on time’, according to the institutional rhythm, rather than ‘in time’, or spending relational time with women [39]. Maher proposes a middle ground to the experience of time, between linear and cyclical [66]. In keeping with Deery’s depiction of being ‘in time’ with women [39], Maher describes a ‘time in process’, as women appreciate and engage with the embodied ‘forward movement towards the birth of the baby’ (p. 136) [66]. A focus on embodiment may be a way forward and deserves more exploration.

Hill describes how early factories were initially *no more productive* than the cottage industries they replaced, but allowed more control over the labour force. However, as these technologies came to ‘frame’ Western thought about work, it has become impossible to conceive of the organisation of labour outside of an industrial or corporate ‘frame’. This is allegorical to the reframing of birth within the medical model and the way in which science and medicine have constructed women’s bodies as risky; marked out and attempted to eradicate the competing and rival discourse of midwifery; and situated birth in hospitals despite the fact that hospitals were not, to begin with, any safer [34, 67, 68]. Industrialized birth has therefore become the frame through which childbirth is perceived.

Critique of the industrial nature of maternity institutions is not new; however, it’s identification as a potential *risk* to women is relatively recent. It came to global attention in late 2014 when the National Institute for Health and Care Excellence (NICE) updated guidelines for antenatal care recommended that women in labour be informed of the increased risk of intervention when birthing in an obstetric-led labour ward compared to a midwifery-led unit [69] following the results of the UK Birthplace study [70]. Although our research is not generalisable, and cannot identify definitive causes, it does give a deep, localised account of hospital birth culture and in this sense the knowledge gained is transferable; providing qualitative support to the extant quantitative data which show that medical settings may no longer be adequate as the primary birthplace for the majority of women. In addition, in our attempt to trouble ideas of risk and safety and the relationship of the institution to normal birth, we have focused on a particular view of the institution; one that we hope is understood as a way towards re-visioning birth rather than a simplistic critique of hospital birth *per se*.

## Conclusion

In this article we have delineated the cultural setting of a hospital labour ward and shown how the institutional ‘framing’ of birth, with its risk-orientation, reliance on technology, and medical understanding of birth as something that requires ‘fixing’ influenced the hospital birth apparatus. The mechanics of this apparatus were upheld by a panoptic disciplinary power, *Organisational technology*—symbolised by the *Journey Board—*that served to maintain an institutional momentum within which women and midwives were expected to conform. This momentum was recognized by midwives and doctors as having its own impetus and they practised in ways which sometimes resisted it and at other times surrendered to it. While the hospital managed actual complications very well, and this is not to be downplayed, it had a contradictory effect on normal, physiological birth; the momentum of the institution potentially placed women at risk by ignoring both birth physiology and women’s individual needs, while purportedly upholding their safety, revealing an inherent institutional paradox which mirrors the risk/safety paradox in the wider medical discourse.

The intermediate-social level analysis described in this article situates the institution as a formidable conductor of dominant birth discourse, with a strong surveillance apparatus in place. The *Paradox of the institution* identifies the hospital labour ward as a setting that can place women at unnecessary yet covert risk, and provokes questions as to the relevance and safety of institutional birth in the postmodern age. The implementation of alternative childbirth settings, such as freestanding, midwifery-led birth centres should be considered a matter of priority.

## Abbreviations

ARM: Artificial rupture of membranes
CMA: Critical medical anthropology
CTG: Cardiotocograph
GBS: Group B streptococcus
GD: Gestational diabetes
IOL: Induction of labour
LSCS: Lower segment caesarean section
MET: Medical emergency team
SOL: Spontaneous onset of labour
SRM: Spontaneous rupture of membranes
Synt/o: Syntocinon
T/L: Team leader
VE: Vaginal examination

## References

[1] Jordan B (1993). Birth in four cultures: a cross cultural investigation of childbirth in Yucatan, Holland, Sweden, and the United States.

[2] Davis-Floyd R, Sargent C (1997). Childbirth and authoritative knowledge: cross-cultural perspectives.

[3] Davis-Floyd R (1994). The technocratic body: American childbirth as cultural expression. Social Science and Medicine.

[4] Behruzi R, Hatem M, Goulet L, Fraser W, Misago C (2013). Understanding childbirth practices as an organizational cultural phenomenon: a conceptual framework. BMC Pregnancy and Childbirth.

[5] Cooper G, MacArthur C, Wilson M, Moore P, Shennan A (2010). Satisfaction, control and pain relief: short— and long-term assessments in a randomised controlled trial of low-dose and traditional epidurals and a non-epidural comparison group. International Journal of Obstetric Anesthesia.

[6] Anim-Somuah M, Smyth R, Howell C. Epidural versus non-epidural or no analgesia in labour. Cochrane Database Syst Rev. 2011; Issue 12. Art. No.: CD000331. doi:10.1002/14651858.CD000331.pub3. 10.1002/14651858.CD000331.pub2 16235275

[7] Lieberman E, O'Donoghue C (2002). Unintended effects of epidural analgesia during labor: a systematic review. American Journal of Obstetrics and Gynecology.

[8] Ramin S, Gambling D, Lucas M, Sharma S, Sidawi J, Leveno K (1995). Randomized trial of epidural versus intravenous analgesia during labor. Obstetrics and Gynecology.

[9] Gaiser R (2005). Labor epidurals and outcome. Best Practice & Research Clinical Anaesthesiology.

[10] Tracy S, Sullivan E, Wang Y, Black D, Tracy M (2007). Birth outcomes associated with interventions in labour amongst low risk women: a population-based study. Women and Birth.

[11] Jordan S, Emery S, Watkins A, Evans J, Storey M, Morgan G (2009). Associations of drugs routinely given in labour with breastfeeding at 48 h: analysis of the Cardiff births survey. BJOG.

[12] Wang F, Shen X, Guo X, Peng Y, Gu X (2009). Epidural analgesia in the latent phase of labor and the risk of Cesarean delivery. Anesthesiology.

[13] Wiklund I, Norman M, Uvnäs-Moberg K, Ransjo-Arvidson AB, Andolf E (2009). Epidural analgesia: breast-feeding success and related factors. Midwifery.

[14] Rahm V, Hallgren A, Hogberg H, Hurtig I, Odlind V (2002). Plasma oxytocin levels in women during labor with or without epidural analgesia: a prospective study. Acta Obstetricia et Gynecologica Scandinavica.

[15] Walsh D (2009). Pain and epidural use in normal childbirth. Evidence Based Midwifery.

[16] Newnham E, Pincombe J, McKellar L (2016). Critical medical anthropology in midwifery research: a framework for ethnographic analysis. Global Qualitative Nursing Research.

[17] Downe S (2008). Normal childbirth: evidence and debate.

[18] Fahy K, Hastie C, Fahy K, Foureur M, Hastie C (2008). Midwifery guardianship: reclaiming the sacred in birth. Birth Territory and midwifery Guardianship: Theory for Practice, Education and Research.

[19] Walsh D (2012). Evidence and skills for normal birth.

[20] Davis-Floyd R (1992). Birth as an American rite of passage.

[21] Rothman BK (1989). Recreating motherhood: ideology and technology in a patriarchal society.

[22] Martin E (1989). The woman in the body.

[23] Oakley A (1984). The captured womb: a history of the medical care of pregnant women.

[24] Baer H, Singer M, Johnsen JH (1986). Toward a critical medical anthropology. Social Science & Medicine.

[25] Newnham EC, McKellar LV, Pincombe JI (2015). Documenting risk: a comparison of policy and information pamphlets for using epidural or water in labour. Women and Birth.

[26] Ezzy D. Coding data and interpreting text: methods of analysis. In: Qualitative Analysis: Practice and innovation. Sydney: Allen and Unwin; 2002. p. 80–110.

[27] Singer M, Baer H (1995). Critical medical anthropology.

[28] Thomas J (1993). Doing critical ethnography.

[29] Hammersley M, Atkinson P (1983). Ethnography: principles in practice.

[30] Geertz C (1973). Thick description: toward an interpretive theory of culture. The interpretation of cultures: selected essays.

[31] Morse J (2006). The politics of evidence. Qualitative Health Research.

[32] Sandelowski M (1986). The problem of rigor in qualitative research. Advances in Nursing Science.

[33] Navarro V (1986). Crisis, Health and Medicine: A Social Critique.

[34] Murphy-Lawless J (1998). Reading birth and death: a history of obstetric thinking.

[35] Foucault M (2003). The birth of the clinic [1963].

[36] Willis E (1989). Medical dominance.

[37] Waitzkin H (1983). The second sickness: contradictions of capitalist health care.

[38] Odent M (1999). The scientification of love.

[39] Deery R (2008). The tyranny of time: tensions between relational and clock time in community-based midwifery. Social Theory & Health.

[40] Dykes F (2009). Applying critical medical anthropology to midwifery research. Evidence Based Midwifery.

[41] Szurek J, Davis-Floyd R, Sargent C (1997). Resistance to technology-enhanced childbirth in Tuscany: The political economy of Italian birth. Childbirth and authoritative knowledge: cross-cultural perpectives.

[42] Foucault M (2008). The history of sexuality: volume 1 [1976].

[43] Foucault M, Gordon C (1980). Two lectures. Power/knowledge: selected interviews and other writings 1972–1977 by Michel Foucault.

[44] Foucault M, Burchell G, Gordon C, Miller P (1991). Politics and the study of discourse. The Foucault Effect: Studies in Governmentality.

[45] Foucault M (1991). Discipline and punish: the birth of the prison.

[46] Walsh D (2006). Subverting the assembly-line: childbirth in a free-standing birth centre. Social Science & Medicine.

[47] Blaaka G, Eri T (2008). Doing midwifery between different belief systems. Midwifery.

[48] Behruzi R, Hatem M, Fraser W, Goulet L, Ii M, Misago C (2010). Facilitators and barriers in the humanization of childbirth practice in Japan. BMC Pregnancy and Childbirth.

[49] Kirkham M, Kirkham M (2000). How can we relate?. The midwife-mother relationship.

[50] Kirkham M, Stapleton H. The culture of the maternity services in Wales and England as a barrier to informed choice. In: Kirkham, M, editor. Informed Choice in Maternity Care. Houndmills, Basingstoke: Palgrave MacMillan; 2004.

[51] Hunt S, Symonds A (1995). The social meaning of midwifery.

[52] Dykes F (2005). A critical ethnographic study of encounters between midwives and breast-feeding women in postnatal wards in England. Midwifery.

[53] Arney WR (1982). Power and the profession of obstetrics.

[54] Downe S, Dykes F, McCourt C (2009). Counting time in pregnancy and labour. Childbirth, midwifery and concepts of time.

[55] Stevens T, McCourt C (2009). Time and midwifery practice. Childbirth, Midwifery and Concepts of Time.

[56] O’Connell R, Downe S (2009). A metasynthesis of midwives’ experience of hospital practice in publicly funded settings: compliance, resistance and authenticity. Health.

[57] Chaboyer W, Wallen K, Wallis M, McMurray A (2009). Whiteboards: one tool to improve patient flow. Medical Journal of Australia.

[58] O’Brien L, Bassham J, Lewis M (2015). Whiteboards and discharge traffic lights: visual management in acute care. Australian Health Review.

[59] Davis-Floyd R, Davis E, Davis-Floyd R, Sargent C (1997). Intuition as authoritative knowledge in midwifery and home birth. Childbirth and authoritative knowledge: cross-cultural perspectives.

[60] Winter C, Duff M, McCourt C (2009). The progress of labour: orderly chaos?. Childbirth, midwifery and concepts of time.

[61] Walsh D, McCourt C (2009). Management of time and place in a birth centre. Childbirth, midwifery and concepts of time.

[62] Kirkham M, Byrom S, Downe S (2015). How environment and context can influence capacity for kindness. The roar behind the silence.

[63] Walsh D (2010). Childbirth embodiment: problematic aspects of current understandings. Sociology of Health & Illness.

[64] Hill S (1988). The tragedy of technology.

[65] McCourt C, Dykes F, McCourt C (2009). From traditional to modernity: Time and childbirth in historical perspective. Childbirth, midwifery and concepts of time.

[66] Maher J (2008). Progressing through labour and delivery: birth time and women’s experiences. Women’s Studies International Forum.

[67] Tew M (1990). Safer childbirth: a critical history of maternity care.

[68] Newnham EC (2014). Birth control: power/knowledge in the politics of birth. Health Sociology Review.

[69] NICE. Intrapartum care: care of healthy women and their babies during childbirth [CG190]. National Institute for Health and Care Excellence; 2014. 25950072

[70] Brocklehurst P, Hardy P, Hollowell J, Linsell L, Macfarlane A, McCourt C, Marlow N, Miller A, Newburn M, Petrou S (2011). Perinatal and maternal outcomes by planned place of birth for healthy women with low risk pregnancies: the birthplace in England national prospective cohort study. BMJ.

